# Red ginseng (*Panax ginseng* Meyer) oil: A comprehensive review of extraction technologies, chemical composition, health benefits, molecular mechanisms, and safety

**DOI:** 10.1016/j.jgr.2021.12.006

**Published:** 2021-12-17

**Authors:** Van-Long Truong, Woo-Sik Jeong

**Affiliations:** Food and Bio-industry Research Institute, School of Food Science & Biotechnology, College of Agriculture and Life Sciences, Kyungpook National University, Daegu, Republic of Korea

**Keywords:** *Panax ginseng*, Red ginseng oil, Health benefits, Molecular mechanism, Safety

## Abstract

Red ginseng oil (RGO), rather than the conventional aqueous extract of red ginseng, has been receiving much attention due to accumulating evidence of its functional and pharmacological potential. In this review, we describe the key extraction technologies, chemical composition, potential health benefits, and safety of RGO. This review emphasizes the proposed molecular mechanisms by which RGO is involved in various bioactivities. RGO is mainly produced using organic solvents or supercritical fluid extraction, with the choice of method greatly affecting the yield and quality of the end products. RGO contains a high unsaturated fatty acid levels along with considerable amounts of lipophilic components such as phytosterols, tocopherols, and polyacetylenes. The beneficial health properties of RGO include cellular defense, antioxidation, anti-inflammation, anti-apoptosis, chemoprevention, hair growth promotion, and skin health improvement. We propose several molecular mechanisms and signaling pathways that underlie the bioactivity of RGO. In addition, RGO is regarded as safe and nontoxic. Further studies on RGO must focus on a deeper understanding of the underlying molecular mechanisms, composition–functionality relationship, and verification of the bioactivities of RGO in clinical models. This review may provide useful information in the development of RGO-based products in nutraceuticals, functional foods, and functional cosmetics.

## Graphical abstract

**Figure:**
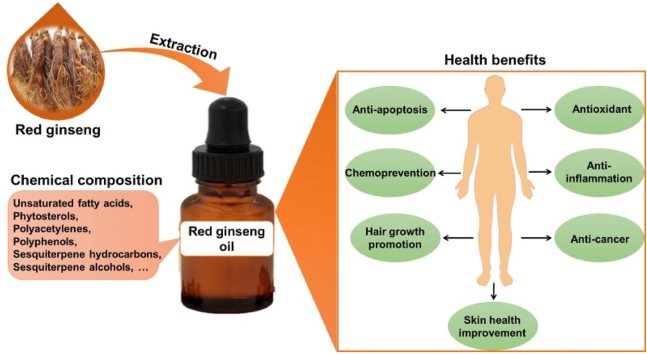

## 1 Introduction

Korean ginseng (*Panax ginseng* Meyer) is native to the mountainous regions of East Asia and has a long-recorded history of use in folk medicine applications for restoration and healing the human body and preventing disease. Ginseng is also known to be an adaptogen, capable of stabilizing physiological functions and enhancing body strength to resist various environmental and emotional stress conditions [1]. In recent years, the biological properties of ginseng have been extensively demonstrated, including antioxidant, anti-inflammation, anti-cancer, anti-fatigue, and anti-aging functions, as well as the ability to boost the immune system, enhance memory, and improve blood circulation [[1], [2], [3], [4], [5], [6], [7], [8]].

As the shelf life of fresh ginseng is short, the development of post-harvesting and long-term storage methods is required to prevent degradation due to temperature, moisture, bacteria, and fungi. In Korea, two traditional preparation methods have been developed to address these issues. The simplest processing method is dehydration of ginseng root by sun-drying, which generates white ginseng. Over time an alternative processing procedure was developed, in which the ginseng root is steamed for 1–3 h at 90–98 °C, followed by sun-drying to obtain a moisture content of 15%–18% [9,10]. A Maillard reaction occurs during the process of steaming and drying, turning the ginseng a reddish or brown hue from which the name “red ginseng” originates [11]. These traditional processing methods not only extend shelf life, but may also have other impacts such as removing unpleasant smell, reducing toxic or undesired side effects, modifying chemical composition, and enhancing biological benefits [9].

Originally, the production of red ginseng was intended to improve the shelf life of ginseng for long-term storage. However, different processing methods resulted in alterations to the chemical profiles and pharmaceutical properties of ginseng [9,10]. Furthermore, red ginseng has greater biological benefits and fewer side effects compared with fresh and white ginseng [9,10,12,13]. Through extensive research, the biological activities of Korean ginseng as well as its processed ginseng products have been attributable to various functional components including ginsenosides, polyacetylenes, phenolic compounds, alkaloids, polysaccharides, oligopeptides, and essential oil [4,[14], [15], [16]].

Once thought to be only a waste product, red ginseng marc is currently considered to be an exclusive source of bioactive constituents for applications in pharmacy, nutrition, and cosmetics [[17], [18], [19]]. Previously, the lipophilic fraction of red ginseng has attracted less attention and was often discarded due to its small quantity. However, several pieces of evidence have demonstrated the importance of the lipid-soluble fraction of ginseng, which confers many health benefits [20,21]. Thus, modern studies have focused on investigating the composition of red ginseng oil (RGO) and the health benefits derived from its consumption.

RGO, the subject of this review, is a group of lipid-soluble phytochemicals or bioactive compounds found in red ginseng marc that have been gaining attention as a health-promoting dietary component. RGO possesses a good fatty acid profile along with other phytochemicals such as phytosterols, polyphenols, tocopherols, and polyacetylenes, which are thought to be responsible for the health benefits of RGO. RGO can be mass-produced at low cost and developed into commercial products for human consumption. Over the past decade, attempts have been made to extract oil from red ginseng and to explore its biological activities. However, RGO has not received sufficient attention. This review summarizes the extraction techniques for RGO, its chemical composition, and the health benefits that underlie the potential food, nutraceutical, cosmetic, and pharmaceutical applications of RGO.

## 2 Extraction of RGO

**Fig. 1:**
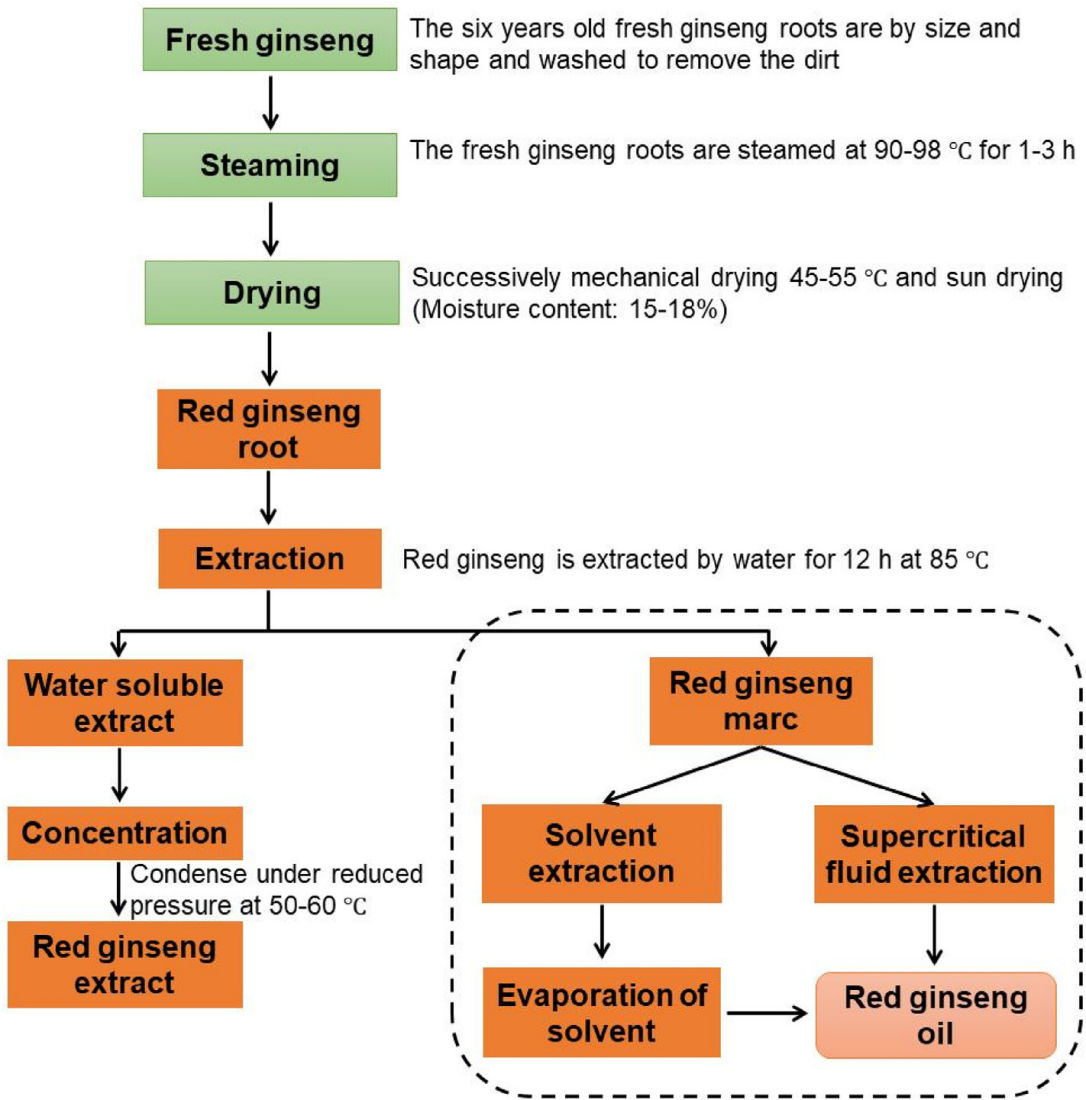
Production methods for red ginseng extract and red ginseng oil. Fresh ginseng (6 years old) is washed, steamed, and dried before extraction of the waster-soluble extract. Red ginseng marc, a by-product of water-soluble extract production, is the source material for red ginseng oil, which is extracted using either a solvent or with the supercritical fluid method. Modified from Lee and colleagues [10].

Extraction technologies are developed to optimize the yield and quality of oil while minimizing production costs. Moreover, it is important that the natural proportion of components in the starting material is maintained during extraction by any technology employed [22]. There are a few well-documented procedures for extracting ginseng oil, including conventional solvent extractions and supercritical fluid extraction (Fig. 1).

### 2.1 Conventional extractions

Solvent extraction is a conventional method for plant oil production due to its relatively low cost and high extraction efficiency. Hexane is widely used for RGO extraction due to its polarity [[23], [24], [25]]. However, hexane exhibits only a moderate capacity to extract both fatty acids and minor components from solid materials [26]. Other solvents such as petroleum ether have been used to extract RGO [27,28]; however, the use of these solvents is not commercially feasible due to their toxicity. It is impossible to extract all of the desired components using a single solvent, due to the fact that red ginseng contains a diverse array of lipid-soluble components of different polarities. In addition, conventional hydrodistillation and steam distillation can be used to extract red ginseng essential oil. Although conventional extraction methods are commercial and easy to scale up, they are time-consuming and possess disadvantages such as low yield, loss of valuable compounds, contamination with toxic solvent residues, and formation of undesired byproduct [[29], [30], [31]].

### 2.2 Supercritical fluid extraction

Supercritical fluid extraction (SFE) of RGO is an efficient alternative to the conventional methods discussed above. In SFE, red ginseng marc is loaded into extraction vessel and supercritical fluid is added at a specific flow rate until the appropriate extraction conditions are achieved. Supercritical solvent fluid containing the dissolved materials is then moved to a separator at a lower pressure, where the RGO is collected, and the solvent fluid can be recycled. The use of CO_2_ as a solvent in SFE has many advantages such as low critical temperature, low cost, lack of toxicity, lack of odor and taste, non-flammability, environmental friendliness, and ease of residue-free solvent removal [22,32]. In addition, supercritical CO_2_ fluid has the properties of a good solvent in that it rapidly penetrates a solid matrix and effectively dissolves various nonpolar and select polar molecules [29,32]. The extraction process is performed at a low temperature to minimize the degradation of thermally sensitive compounds [33], and a product with a desired composition can be selectively extracted by varying the extraction conditions [22,34]. Furthermore, the CO_2_ is recycled, and the extraction process is repeated many times, increasing extraction efficacy and reducing product cost. Advantageously, the SFE system can be applied from analytical to industrial scales.

Studies in our laboratory show that total unsaturated fatty acids and minor components are present at higher levels in RGO obtained by supercritical CO_2_ fluid extraction than in RGO extracted using conventional procedures (*unpublished data*). These data are in agreement with another study that indicated that phytosterol content in ginseng seed oil obtained by supercritical CO_2_ fluid extraction was greater than in that obtained by conventional methods such as solvent extraction and compression extraction [26]. Fermented ginseng seed oil obtained by supercritical CO_2_ fluid extraction also showed significantly higher phenolic compound and phytosterol contents compared with that obtained by compression extraction or solvent extraction [35]. Therefore, SFE using CO_2_ appears to be the optimum extraction method for obtaining RGO.

## 3 Chemical composition of RGO

RGO contains fatty acids, phytosterols, hydrocarbons, polyacetylenes, polyphenols and many other components. RGO is uniquely rich in unsaturated fatty acids, which account for approximately 80–90% of its total fatty acids [27,36,37]. The level of unsaturated fatty acids in RGO is comparable to that in Korean fermented ginseng seed oil and in American ginseng (*Panax quinquefolius* L.) seed oil. However, the predominant unsaturated fatty acid in RGO is the polyunsaturated linoleic acid, while a monounsaturated fatty acid, oleic acid, is predominant in both Korean and American ginseng seed oils [35,37,38]. In addition, RGO contains significant amounts of oleic acid, linolenic acid, and *cis*-11,14-eicosatyrienoic acid, and also contains other unsaturated fatty acids such as erucic acid, *cis*-13,16-docosadienoic acid, nervonic acid, *cis*-11-eicosenoic acid, palmitoleic acid, gamma-linolenic acid, arachidonic acid, and *cis*-10-heptadecenoic acid [27,37]. RGO also contains saturated fatty acids such as palmitic acid, stearic acid, pentadecanoic acid, lignoceric acid, heptadecanoic acid, arachidic acid, tricosanoic acid, behenic acid, myristic acid, and heneicosanoic acid; of these, palmitic acid is the most prevalent [37].

Polyphenols are a group of diverse compounds that exert multiple biological functions such as antioxidant, anti-inflammation, and cancer prevention. Red ginseng contains a wide variety of phenolic compounds including maltol, salicylic acid, vanillic acid, p-coumaric acid, ferulic acid, p-hydroxybenzoic acid, gentisic acid, and cinnamic acid [[39], [40], [41]]. The total polyphenol content in RGO was 243 mg of tannic acid equivalents per 100 g RGO. However, the precise composition and characteristics of the phenolic contents in RGO remain unknown.

Importantly, RGO is a rich source of phytosterols, containing approximately 108 mg phytosterols per g RGO. The most abundant phytosterol in RGO is β-sitosterol (90.3 mg/g), followed by stigmasterol (13.7 mg/g) and campesterol (3.9 mg/g) [42]. High-performance liquid chromatography analysis of ethanolic extracts of ginseng root and processed ginseng products (i.e., straight ginseng, white ginseng, and red ginseng) showed the presence of β-sitosterol and stignasterol; six-year-old red ginseng was found to have a β-sitosterol and stignasterol content of 21.21 mg/g and 6.24 mg/g, respectively [43]. RGO also contains α-tocopherol, which is the most active known form of vitamin E and is necessary for antioxidant function, protection of the cellular membrane, regulation of platelet aggregation, and prevention of disease [44,45].

Hydrocarbons such as bicycle (10.1.0)tridec-1-ene, stigmastan-3,5-diene, and tritetracontane can be found in RGO [36]. Red ginseng essential oil also contains sesquiterpene hydrocarbons, sesquiterpene alcohols, monoterpenes, aldehydes, esters, acids, and other miscellaneous compounds. The distinctive aroma of ginseng and red ginseng is produced by the presence of β-caryophyllene, β-panasinsene, bicyclogermacrene, aneoclovene, α-panasinsene, spathulenol, ginsenol, selina-4,11-diane, and neointernedeol [[46], [47], [48], [49]]. To date, there have been almost no studies on the physiological activity of these aroma-related volatile compounds; however, these volatiles are an important element in the sensory quality of ginseng. Red ginseng is strongly fragrant, with sweet and roast flavor notes, which is attributable to the aroma-related compound 3-hydroxy-2-methyl-pyran-4-one [50].

Polyacetylenes are representative water-insoluble substances from ginseng. To date, about 20 polyacetylene compounds have been identified in ginseng [10,39]. RGO extracted using various solvents has been found to contain polyacetylenes such as panaxydol, panaxynol, and panaxytriol, of which panaxydol and panaxynol account for over 90% of the total polyacetylene content. In addition, RGO also contains other polyacetylenes such as heptadeca-1-ene-4,6-diyn-3,9-diol, panaxyne, panaxyne epoxide, acetylpanaxydol, 10-acetylpanaxytriol, and ginsenoynes [20,[51], [52], [53]]. Panaxytriol, a hydrated compound with an epoxy ring, is unique to Korea red ginseng and is converted from panaxydol by the heat and acidity present during red ginseng preparation [4,10]. These polyacetylenes exert multiple physiological activities including memory improvement, antioxidant, anti-inflammatory, and hypocholesterolemic properties as well as suppression of lipid peroxidation, cancer cell proliferation, and genetic mutations and oncogenesis. In particular, anti-cancer activity of RGO may be attributable to polyacetylene compounds [21,24,54,55]. However, due to their chemical instability and tendency to undergo oxidation, the therapeutic characteristics of ginseng polyacetylenes require further evaluation.

## 4 Biological activities of RGO and their underlying mechanisms

As outlined above, RGO contains numerous functional compounds such as unsaturated fatty acids, phytosterols, polyphenols, and polyacetylenes, which provide health benefits. In this section, we expand upon the available evidence for the biological activities of RGO as well as its mechanisms of action.

### 4.1 Antioxidant properties

RGO contains a considerable amount of various phytochemicals with antioxidant potential, suggesting an ability to provide protection from unfavorable effects of oxidative/nitrosative stress in the human body. Free radical scavenging activity is generally achieved through hydrogen atom transfer and/or electron transfer mechanisms, and many *in vitro* chemical assays based on these mechanisms have been developed to examine antioxidant activity of phytochemicals or their derivatives [56]. RGO was shown to scavenge peroxyl radicals in the oxygen radical absorbance capacity (ORAC) assay, with 0.1% RGO obtaining an ORAC value of 0.78 μM Trolox equivalent [57]. It was also found that RGO at dilutions between 0.01% and 0.1% exhibited a dose-dependent increase in reducing ability in the cupric-reducing antioxidant capacity assay. Another study reported antiradical activity of RGO in the 2,2-diphenyl-1-picrylhydrazyl (DPPH) radical scavenging assay, where the radical scavenging capacity of 1% RGO was equivalent to 70% α-tocopherol; this was attributed to a great number of available hydroxyl groups in RGO, as evident from a total polyphenolic content of 243 mg tannic acid equivalents per 100 g RGO [58]. These results suggest that phytochemicals in RGO exert potent free radical scavenging activity through hydrogen atom and electron transfer mechanisms.

Chemical-based antioxidant activity assays, as described above, provide preliminary evidence for antioxidant properties in a plethora of phytochemicals. However, each assay only reflects antioxidant activity or chemical reactivity of a substance under those specific assay conditions, and is unlikely to fully represent total antioxidant capacity within the complexity of biological systems [59]. Antioxidative reactions *in vivo* may occur via multiple mechanisms and depend upon a variety of factors. Moreover, some chemical-based assays lack *in vivo* relevance as they are not performed at physiological pH and temperature; more importantly, no chemical-based assay takes into account the absorption, bioavailability, metabolism, and excretion of antioxidant compounds [60,61]. Thus, cell-based antioxidant assays have been developed to address some of these issues. RGO effectively attenuated oxidative stress in HepG2 cells induced by the radical initiators 2,2-azobis (2- amidino-propane) dihydrochloride and Cu^2+^ ion [57]. Previous studies in our laboratory showed that RGO significantly reduced intracellular ROS production in H_2_O_2_-treated HepG2 cells [62] and in 12-O-tetradecanoylphorbol-13-acetate (TPA)-stimulated JB6 tumor-promotion-sensitive (P+) mouse epidermal cells [63]. RGO was shown to strongly inhibit amyloid beta peptide (Aβ_25–35_)-induced intracellular ROS production in PC12 cells, suggesting the ability to confer protection against Aβ_25–35_-induced oxidative damage and neurotoxicity [64]. Furthermore, the neuroprotective effect of RGO against Aβ_25–35_-induced oxidative stress was associated with its major constituents such as linoleic acid, β-sitosterol, and stigmasterol [65]. Although these studies did not establish the precise antioxidant mechanisms, it is reasonable to presume that phytochemicals in RGO may act directly by scavenging free radicals and/or indirectly by enhancing cellular antioxidant defense systems.

In addition to the direct antioxidant capacity of RGO, recent studies have demonstrated that RGO acts as an indirect antioxidant via upregulating primary antioxidant enzymes. In our previous work, treatment with RGO was able to maintain normal protein level and activity of CAT, SOD2, and GPx in H_2_O_2_-treated HepG2 cells [62]. In the same study, the indirect antioxidant capacity of RGO (10 and 50 mg/kg) was also demonstrated in a mouse model of carbon tetrachloride (CCl_4_)-induced hepatotoxicity. The metabolism of CCl_4_ by microsomal cytochrome P450 in the liver generates highly reactive oxidants that cause lipid peroxidation and, ultimately, liver injury as detected by significantly increases in thiobarbituric acid-reactive substance value, serum aspartate transaminase (AST) and alanine transaminase (ALT); however, oral administration of RGO mitigated CCl_4_-induced liver injury by reducing hepatic lipid peroxidation and restoring the activity and protein expression level of CAT, SOD2, and GPx. Similarly, in a model of azoxymethane/dextran sodium sulfate (AOM/DSS)-induced inflammation-related colon carcinogenesis, we confirmed that RGO (100 mg/kg) contributed to the protection of the colon from oxidative stress by decreasing the colonic level of malondialdehyde and increasing the expression and activity of antioxidant enzymes [66]. A role of RGO in regulating endogenous antioxidant enzymes and preventing aging in the skin has also been demonstrated [67]. The skin is one of the few organs that comes into direct contact with exogenous ROS/RNS sources, such as UV light; despite the fact that the skin is equipped with complex defense systems involving UV absorption, DNA repair, and ROS detoxification, these defense mechanisms may be overwhelmed by prolonged UV exposure or high-energy UV radiation. In a recent study, we also found that skin injury was induced by UVC radiation, which causes large quantities of ROS/RNS production; however, topical application of 1% RGO for three days prevented UVC-induced oxidative damage to lipid and DNA via upregulation of CAT, SOD2, and GPx expression in mouse skin [67]. A recent study have confirmed the protective effects of RGO against acetaminophen-induced hepatotoxicity and UV-induced phototoxicity through enhancing cellular antioxidant defense systems [68].

Overall, the antioxidant properties of RGO have been demonstrated in chemical-based, *in vitro*, and *in vivo* assays. It is thought that the antioxidant capacity of RGO is attributable to its phytochemicals; however, the *in vivo* effects of these compounds have not been sufficiently studied. Thus, further *in vivo* studies on the antioxidant effects of the bioactive components in RGO, particularly at physiologically relevant concentrations, are necessary.

### 4.2 Anti-inflammatory properties

**Fig. 2:**
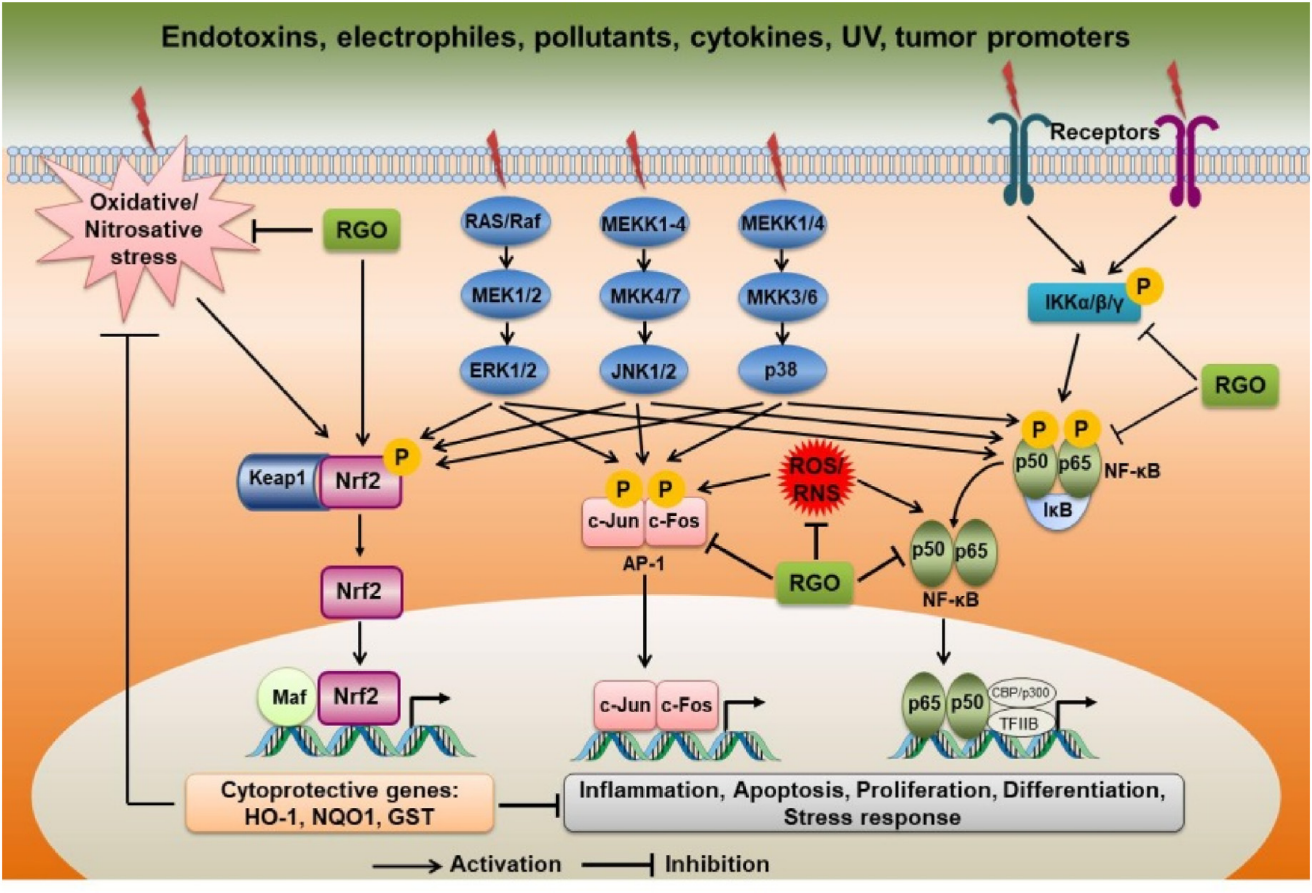
Possible mechanisms underlying the antioxidant, anti-inflammatory, and chemopreventive properties of red ginseng oil. Multiple external and internal stimuli triggers signal transduction cascades that converge on and rapidly activate transcription factors such as NF-κB and AP-1. Consequently, this leads to transcriptional expression of target genes such as pro-inflammatory cytokines, chemokines, and intracellular adhesion molecules involved in inflammation, cellular proliferation, neoplastic transformation, and tumor promotion. Red ginseng oil can inhibit the activation of NF-κB and AP-1 through down-regulating singling pathways. Nrf2 is a master transcription factor that regulates a wide range of phase II detoxification and antioxidant enzymes such as NAD(P)H:quinone oxidoreductase (NQO1), heme oxygenase-1 (HO-1), and glutathione S-transferase (GST). Red ginseng oil can activate Nrf2 pathway, thereby enhancing cytoprotective system, reducing the level of oxidative stress, and interfering with NF-κB and AP-1 activation.

Inflammation is mediated by interactions between signal transduction pathways, transcription factors, and inflammatory genes (Fig. 2). Many intracellular signal transduction pathways converge on the activation of a distinct set of transcription factors, including nuclear factor κB (NF-κB) and activator protein 1 (AP-1), which act independently or in a coordinated manner to regulate the expression of a large number of target genes [69]. Aberrant activation of NF-κB and AP-1 are associated with inflammation, anti-apoptosis, cell proliferation and differentiation, and neoplastic transformation by driving transcription of inflammatory genes such as inducible nitric oxide synthase (iNOS), cyclooxygenase-2 (COX-2), cytokines, chemokines, and intracellular adhesion molecules [[69], [70], [71], [72]]. In addition, NF-κB and AP-1 are both redox-sensitive transcription factors that are believed to be activated by ROS/RNS; downstream, the genes transcribed by NF-κB and AP-1 activation also encode proteins that contribute to the production of ROS/RNS, such as iNOS, COX-2, arachidonate 12-lipoxygenase, arachidonate 5-lipoxygenase, xanthine oxidoreductase, and NADPH oxidase 2 (gp91^ph^°^x^) [73,74]. Therefore, the suppression of NF-κB and AP-1 is considered to be a promising strategy for the prevention and treatment of a variety of pathological conditions that contain an inflammatory component.

Both *in vitro* and *in vivo* studies have confirmed that anti-inflammatory action of RGO targets the pivotal inflammatory transcription factors NF-κB and AP-1. Our previous study showed that RGO significantly reduced the LPS-induced elevation of iNOS and COX-2, and their corresponding products NO and prostaglandin E2 (PGE2), respectively, in RAW 264.7 macrophages [42]. In the same study, RGO suppressed the LPS-induced activation and nuclear translocation of NF-κB as well as the release of pro-inflammatory cytokines such as interleukin (IL)-1β, IL-6, and TNF-α by inhibiting IKK phosphorylation and preventing proteasomal degradation of IκB. In addition, RGO inhibited the signaling pathway involving transforming growth factor beta (TGF-β)-activated protein kinase 1, MAP kinase 3/6, and p38-MAPK (TAK1−MKK3/6−p38 MAPK pathway), which could account for the ability of RGO to suppress NF-κB activation and expression of NF-κB target genes. Although no studies have yet examined the correlation between the bioactive components of RGO and its anti-inflammatory properties in macrophages, it is reasonable to presume that functional constituents such as unsaturated fatty acids, phytosterols, polyacetylenes, and polyphenols act independently and/or synergistically to produce the anti-inflammatory activity of RGO.

Similarly, in an *in vitro* model of neurotoxicity and neuroinflammation, RGO significantly inhibited Aβ_25–35_-induced activation of NF-κB and MAPK signaling pathways in PC12 cells, leading to the reduced NO and PGE2 production and decreased iNOS, COX-2, and TNF-α expression [64]. More interestingly, compounds found in RGO have been shown to exhibit anti-inflammatory effects in Aβ_25–35_-induced neuroinflammation when tested in their purified forms. Namely, linoleic acid, β-sitosterol, and stigmasterol were found to decrease Aβ_25–35_-induced production of NO and PGE2 as well as expression of iNOS, COX-2, IL-1β, and TNF-α through the suppression of the NF-κB, ERK, JNK, and p38 MAPK signaling pathways in PC12 cells [65]. These data suggest that bioactive components are responsible for the anti-inflammatory properties of RGO. Moreover, RGO has also been shown to inhibit inflammatory signaling pathways in other *in vitro* models: RGO suppressed neoplastic transformation of TPA-stimulated mouse epidermal JB6 P+ cells, with this suppression apparently mediated by blocking activation of NF-κB, AP-1, and MAPK as well as expression of COX-2 [63]; and treatment with RGO inhibited NO production and MAPK signaling during *Brucella abortus* infection in macrophages [75].

*In vivo* studies in mouse models of colitis-associated colon carcinogenesis or UV-exposed skin provide further evidence for the anti-inflammatory activity of RGO. Oral administration of RGO at dose of 100 mg/kg in AOM/DSS-treated mice for 17 weeks significantly attenuated serum levels of NO, IL-6 and TNF-α, as well as expression of colonic inflammatory markers iNOS, COX-2, IL-6, IL-1β, and TNF-α [66]. Further investigations in that study showed that RGO blocked the phosphorylation and subsequent degradation of IκB, thus inhibiting AOM/DSS-induced NF-κB activation. It has also been demonstrated that topical application of RGO decreased skin inflammation in UVC-exposed SKH-1 hairless mice, as evidenced by the reduced level of COX-2 expression; this anti-inflammatory effect may be attributable to blockade of UVC-induced activation of AP-1 and MAPKs [67]. Similarly, our recent study identified that purified forms of compounds found in RGO, such as linoleic acid and β-sitosterol, are also able to suppress UVC-induced activation of AP-1 and MAPKs, indicating inhibition of the inflammatory response in mouse skin (*unpublished data*). Thus, RGO shows anti-inflammatory effects in multiple *in vitro* and *in vivo* models, suggesting a potent agent for nutraceutical application.

### 4.3 Chemopreventive properties

Chemoprevention is a strategy by which carcinogenesis is prevented, either by the inhibition of tumor promotion or progression, or by interference in the initiation of the carcinogenesis process via detoxification of carcinogens or carcinogenic metabolites from exogenous or endogenous sources. One of the most promising strategies of cancer chemoprevention is the induction of cytoprotective proteins. One such group of proteins is phase II enzymes, which are an important component of the cellular defense system involved in neutralizing and eliminating ROS/RNS, and electrophilic and oxidative toxicants, from cells before they are able to damage biomolecules, especially DNA [76].

The transcription factor nuclear factor E2-related factor 2 (Nrf2), a member of the Cap'n’collar family of bZIP proteins, is the master regulator of a battery of antioxidant defense element (ARE)-mediated cytoprotective genes, such as NAD(P)H quinone oxidoreductase 1 (NQO1), heme oxygenase-1 (HO-1), and glutathione S-transferase (Fig. 2). Our previous study showed that RGO induced expression of phase II detoxifying and antioxidant enzymes via Nrf2 activation in HepG2 cells, as evident from the increase in ARE reporter-gene activity and increased protein levels of NQO1 and HO-1 [36]. These upregulations were accompanied by activation of apoptosis signal-regulating kinase 1 (ASK1)-MKK4/7-JNK and p38 MAPK signaling pathway in HepG2 cells. In another study, we also observed RGO enhanced expression of Nrf2 and phase II antioxidant enzyme HO-1 in JB6 P+ cells [63].

The ability of RGO to activate Nrf2 pathway was also confirmed in *in vivo* studies. Oral administration of RGO induced colonic Nrf2 and HO-1 in a model of colitis-associated colon carcinogenesis [66]. In the same study, analysis of gene expression profiles by a cDNA microarray revealed that RGO upregulated expression of the phase II detoxifying genes *Gsta1*, *Gstm3*, *Ugt1a10*, *Sult1c2*, and *Mgst3* in mice, suggesting a colonic protection from carcinogenesis. Additionally, we confirmed that topical application of RGO induced expression of HO-1 in the skin of UVC-exposed SKH 1 hairless mice [67].

### 4.4 Anti-apoptotic properties

**Fig. 3:**
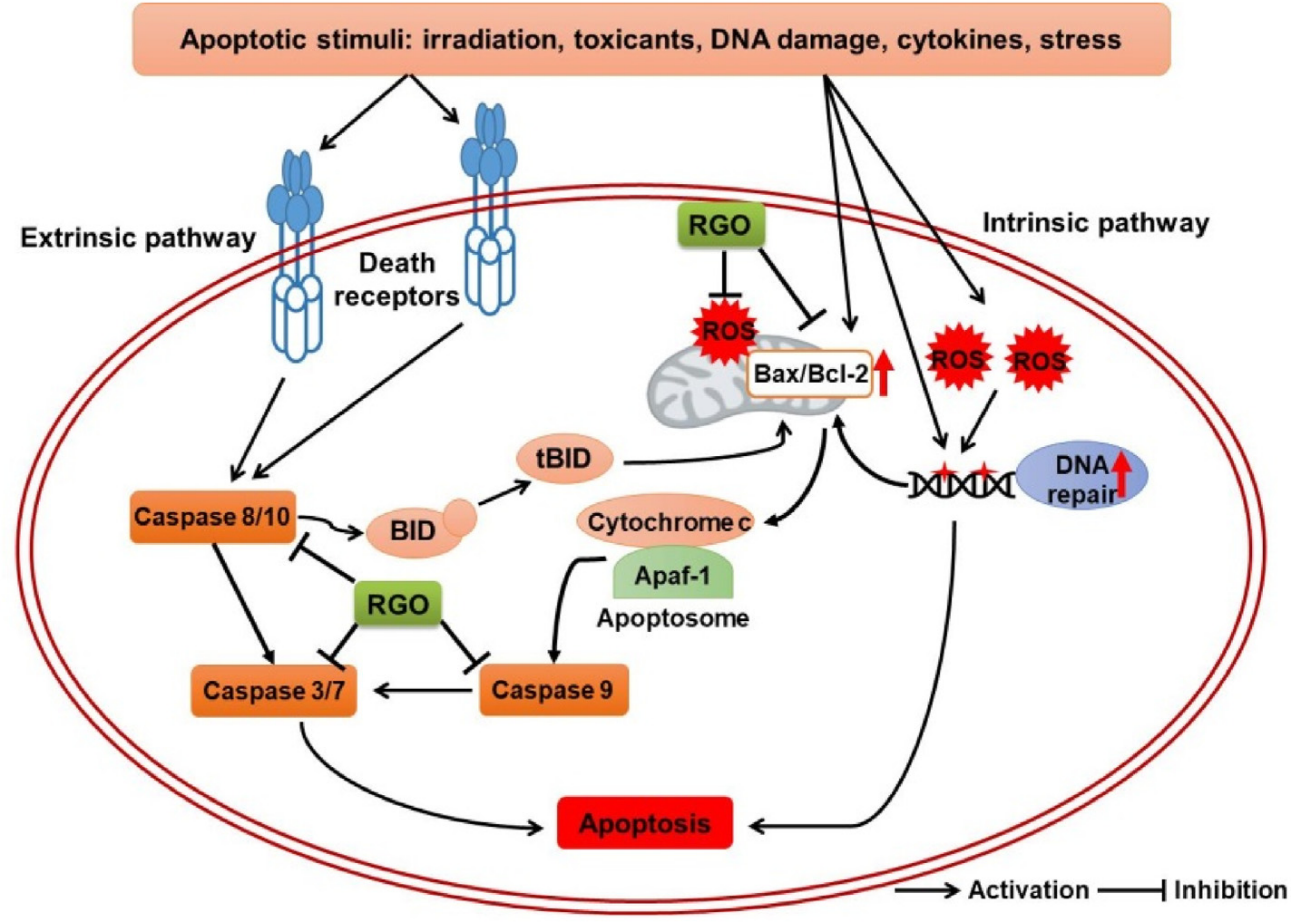
Anti-apoptotic mechanisms of red ginseng oil. Diverse apoptotic stimuli such as cytotoxic insults, DNA-damaging agents, and irradiation trigger extrinsic and intrinsic pathways through activating of cell death receptor and caspase cascades, enhancing mitochondrial membrane permeabilization, and inducing ROS/RNS production and DNA fragmentation. Red ginseng oil exerts protective effect against apoptotic stimuli by inhibiting caspase activation, reducing mitochondrial Bax/Bcl-2 ratio, preventing DNA fragmentation, and suppressing ROS/RNS formation.

Apoptosis, a form of programmed cell death, is the process of eliminating damaged or infected cells. Failure to control apoptosis leads to the development of a large number of different pathologies such as neurodegenerative diseases, cardiovascular diseases, and cancer [77]. While apoptotic processes can be triggered by multiple pathways, the two main and most well-studied pathways are the extrinsic and intrinsic pathways. Several studies have been carried out to evaluate the anti-apoptotic effects of RGO (Fig. 3). Abnormal deposition of Aβ in the brain causes oxidative stress, apoptosis, and neuroinflammation, which contribute to cognitive decline and Alzheimer's disease pathology [78]. Challenge with Aβ_25–35_ decreases cell viability, increases apoptotic cells, and induces G0/G1 cell cycle arrest; however, treatment with RGO or pure forms of its constituent compounds (linoleic acid, β-sitosterol, or stigmasterol) reversed the effects of Aβ_25–35_ in PC12 cells [64,65]. The Aβ_25–35_-induced increased in cytosolic Ca^2+^ levels results in the disruption of membrane Ca^2+^ permeability and subsequent mitochondrial Ca^2+^ overload, which triggers mitochondria-dependent apoptotic cell death. RGO, as well as its major components, inhibited Ca^2+^ influx and maintained mitochondrial membrane potential in Aβ_25–35_-stimulated PC12 cells. In addition, the ratio between pro-apoptotic (Bax) and anti-apoptotic (Bcl-2) proteins involved in the regulation of mitochondrial integrity is regarded as a primary index for determining susceptibility to apoptosis [79]. The Aβ_25–35_-induced increase in the Bax/Bcl-2 ratio in PC12 cells was attenuated by RGO, linoleic acid, β-sitosterol, and stigmasterol [64,65]. In those studies, treatment with RGO or its compounds inhibited Aβ_25–35_-induced activation of caspase 8, 9, and 3, and PARP-1. A study in our laboratory shows that β-sitosterol protects against apoptosis in UVC-exposed HaCaT keratinocytes, as evident from reduced levels of cleaved-caspase 8 and cleaved-PARP (*unpublished data*).

The anti-apoptotic property of RGO was also confirmed in *in vivo* studies. We indicated that topically applied RGO ameliorated apoptosis in the skin by decreasing levels of cleaved caspase-3, cleaved caspase-9, and cleaved PARP, as well as Bax/Bcl-2 ratio in UVC-irradiated SKH1 hairless mice [67]. Similar effects were also observed with topical application of the RGO components linoleic acid and β-sitosterol (*unpublished data*). Additionally, apoptosis in hair follicle is believed to be an important contributor to the process of androgenic hair loss [80]. Hair follicle apoptosis can be induced by androgens such as testosterone and dihydrotestosterone, leading to reduced matrix cell growth, increased hair cell death, early induction of catagen-like transition of hair follicles, and ultimately, hair loss [81,82]. Topical application of RGO, linoleic acid, or β-sitosterol inhibited testosterone-induced apoptosis in a mouse model of androgenic alopecia by decreasing the Bax/Bcl-2 ratio and the level of TGF-β [83]. These data suggest that RGO and its constituent compounds such as linoleic acid, β-sitosterol, and stigmasterol inhibit apoptosis through both intrinsic and extrinsic pathways.

### 4.5 Anti-cancer properties

The global incidence of cancer is increasing due to population growth and aging alongside the increasing adoption of lifestyle choices that increase cancer risk, such as smoking, lack of exercise, and “westernized” diets. The use of dietary nutrients that are capable of inhibiting cell growth and inducing apoptosis has been raised as a potential anti-cancer therapy. To date, several studies reporting on the anti-cancer activity of RGO *in vitro* and *in vivo* have been published.

Treatment of RGO in human lung cancer cells resulted in inhibition of cell growth, G0/G1-phase cell cycle arrest, and induction of apoptosis. A lipid-soluble, n-hexane extract of red ginseng showed a dose-dependent anti-proliferative effect on NCI–H460 human lung cancer cells, with a concentration for 50% growth inhibition (GI_50_) of 81.04 μg/mL [23]. Treatment with this lipid-soluble red ginseng extract at 100 μg/mL for 48 h induced considerable cell cycle arrest at the G0/G1 phase in NCI–H460 cells via down-regulation of G1-phase-related cyclins and cyclin-dependent kinases such as cyclin D−CDK4/6 and cyclin E−CDK2 complexes. The lipid-soluble red ginseng extract significantly increased apoptotic cell death in NCI–H460 cells through activation of caspase cascades, including caspases 3, 8, and 9, in turn leading to cleavage of PARP [20,23]. Lipid-soluble extract of red ginseng exerted potent inhibitory activity on the growth of other cancer cell lines, including small cell lung (DMS114, GI_50_ = 27.0 μg/mL), adenocarcinoma lung (NCI–H23, GI_50_ = 16.7 μg/mL), gastric (MKN74, GI_50_ = 28.2 μg/mL), acute lymphoblastic leukemia (MOLT-4, GI_50_ = 13.7 μg/mL), and prostate (PC-3, GI_50_ = 28.5 μg/mL) cancer cell lines. Treatment with a hexane extract of red ginseng inhibited the proliferation of renal (A498), ovarian (SK-OV-3), glioblastoma (SNB-19), and melanoma (SK-MEL-2) cancer cell lines with GI_50_ values of over 30 μg/mL [84]. Moreover, the anti-cancer activity of RGO was confirmed in *in vivo* mouse models of initial and advanced human lung tumor xenografts without any apparent toxicity [20,84]. In the model examining tumor initiation, oral administration of lipid-soluble red ginseng extract at 0.3 and 1 g/kg/day for 15 days significantly reduced both the volume and the weight of human lung tumor xenografts, which was comparable to 2 mg/kg/day of Adriamycin (doxorubicin), a commonly used anti-cancer drug [20]. In the model examining progression of the tumor after nodule formation, lipid-soluble red ginseng extract suppressed the progression of advanced human lung tumor xenografts [84].

A lipid-soluble red ginseng fraction (petroleum ether extract) exhibited inhibitory effects on growth of three human melanoma cell lines, with the inhibition order SK-MEL-5 > SK-MEL-2 > SK-MEL-1. The inhibitory effect of petroleum ether extract of red ginseng on the growth of these cell lines was due to cell cycle arrest at the G1 phase by upregulation of negative cell cycle regulators such as p27^Kip^ in SK-MEL-2 cells and p21^WAF1^ and retinoblastoma gene product in SK-MEL-1 cells [28]. Hexane extract of red ginseng also exhibited an anti-proliferative effect on B16F10 mouse melanoma cells, with a GI_50_ value of 5.1 μg/mL [24]. The hexane extract of red ginseng strongly suppressed invasion and migration of B16F10 cells even when used at non-cytotoxic concentrations (0.3–3 μg/mL), possibly by downregulating matrix metalloproteinase expression. In the same study, oral administration of hexane extract of red ginseng (1 g/kg/day) for 13 days blocked lung metastasis of B16F10 cells in C57BL/6 female mice, an effect comparable to 2 mg/kg/day of Adriamycin.

Petroleum ether extract of red ginseng was also found to inhibit the growth of and induce cell cycle arrest at G0/G1 phase in three human renal cancer cell lines (Caki-1, A498, and CURC II cells) [85]. In the same study, a purified panaxydol-rich fraction of the petroleum ether extract of red ginseng showed stronger anti-proliferative activity than that of parent extract. More recently, supercritical CO_2_-extracted RGO has been found to inhibit neoplastic transformation in mouse epidermal JB6 P+ cells, as determined by reduced anchorage-independent formation of cell colonies in soft agar [63].

Based on the studies discussed above, RGO shows potential to be considered as preventive and/or therapeutic agent against some types of human cancer. Of note, it was indicated that the lipid-soluble fraction of ginseng possessed a higher anti-cancer activity than that of aqueous ginseng extract, and that the anti-cancer activity of ginseng mainly originate from lipid-soluble components [25]. Polyacetylenes are thought to be the major components of RGO responsible for its anti-cancer activity [20,23,[84], [85], [86]]. The total polyacetylene content in hexane extract of red ginseng was 2.38%–6.88%, with panaxydol, panaxynol, and panaxytriol the dominant polyacetylene species [20]. These polyacetylenes exhibit inhibitory effects on proliferation of cancer cells at low concentration, but show cytotoxicity in healthy cells at much higher concentrations, suggesting a cancer-specific cytotoxic activity [87]. Several studies have examined the anti-proliferative activities of polyacetylene compounds and mechanisms involved [21,85,[87], [88], [89]]. Among the polyacetylenes studied, panaxydol exerts the most potent cytotoxicity against cancer cells [21,39]. Panaxytriol, a polyacetylene unique to Korean Red Ginseng, also exert anti-cancer activity against several cancer cell lines [90,91]. In addition to polyacetylenes, various phytosterol, polyphenolic, and hydrocarbon bioactive compounds may inhibit the growth and proliferation of cancer cells, thereby contributing to anti-cancer property of RGO. It is possible that multiple bioactive compounds in RGO exert synergistic effects, conferring potent anti-cancer activity. Therefore, further studies are required to confirm the anti-cancer properties of RGO and its constituent compounds, and to investigate the underlying molecular mechanisms of its anti-cancer activity.

### 4.6 Hair growth promoting activity

Hair loss, also called alopecia or baldness, refers to a loss of hair from part of the head or body that is not life-threatening but can cause psychological distress. Common types of hair loss include androgenic alopecia, telogen effluvium, chemotherapy-induced alopecia, and alopecia areata. Androgenic alopecia, the most common type of hair loss in humans, occurs in both males and females and is estimated to affect 60%–70% of the worldwide population [92,93]. Androgenic alopecia is characterized by progressive shortening of the anagen phase in successive hair cycles, and follicular miniaturization resulting in the formation of shorter and thinner hair, eventually leading to hair loss [94]. Androgenic alopecia is mediated by the male sex hormone testosterone, which is converted into dihydrotestosterone by 5α-reductase. The androgen receptor in hair cells is activated by binding to androgenic hormones present in the cytoplasm; upon binding, the hormone-receptor complex translocates into the nucleus to drive the transcription of target genes responsible for the negative regulation of hair growth. Although both testosterone and dihydrotestosterone bind to the androgen receptor, dihydrotestosterone binds with a higher affinity than does testosterone [94]. High levels of 5α-reductase and androgen receptor have been detected in the balding scalp of androgenic alopecia patients [95]. A better understanding of the mechanisms of hair growth and alopecia may lead to effective strategies for the prevention and treatment of hair loss.

**Fig. 4:**
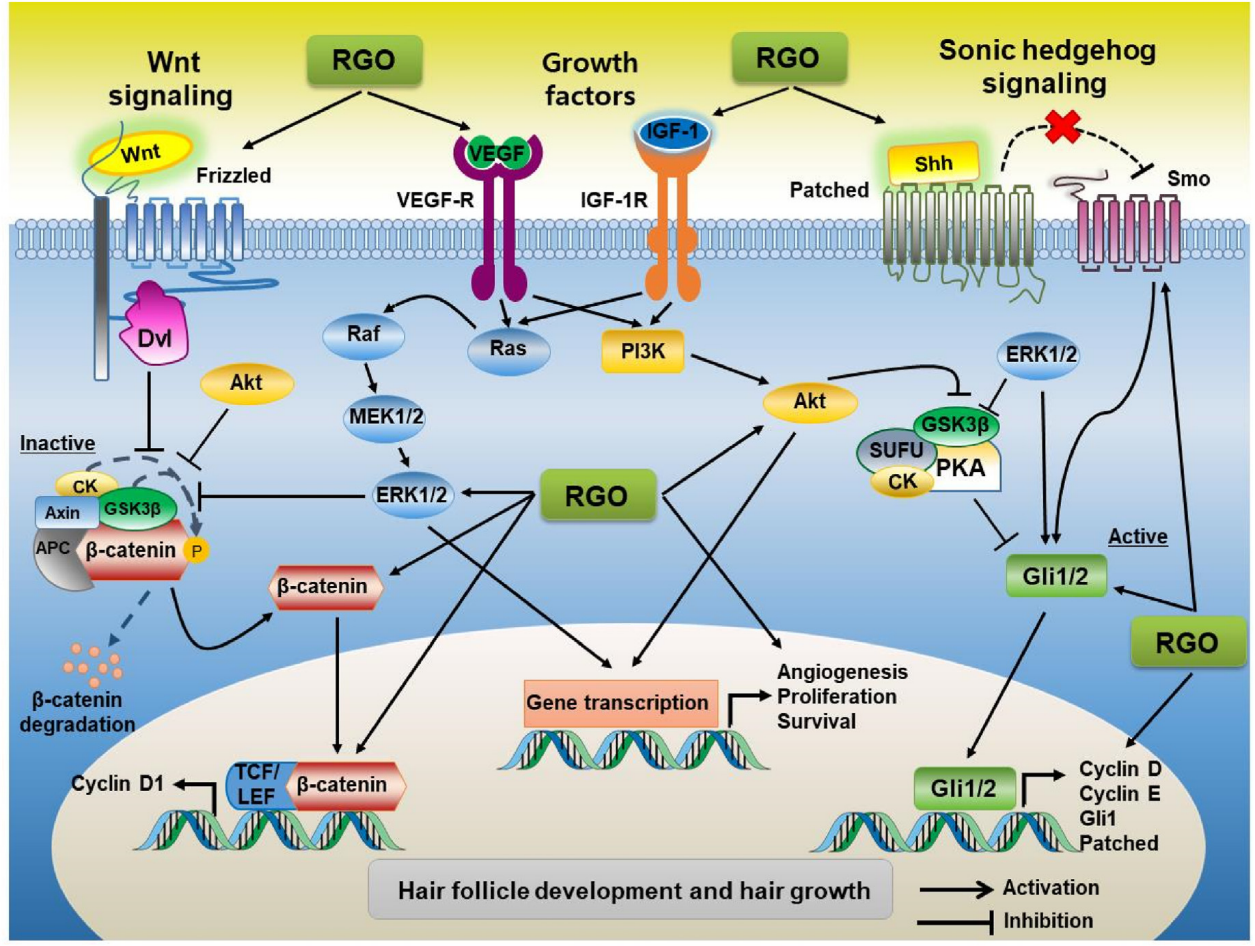
Hair growth promoting activity of red ginseng oil. Cyclic growth of hair is regulated by diverse growth factors and signaling pathways. Red ginseng oil can stimulate hair growth through activating insulin-like growth factor-1 (IGF-1), vascular endothelial growth factor (VEGF), Wnt/β-catenin, and sonic hedgehog signaling pathways, which regulate hair cell proliferation and survival, and maintain the anagen phase.

The ability of RGO to improve hair growth has been demonstrated in *in vitro* and *in vivo* studies (Fig. 4). A study in our laboratory showed that in *in vitro* model, RGO significantly increased the proliferation of human hair dermal papilla cells, which are important contributors to hair formation, growth, and cycling (*unpublished data*). We confirmed that topical application of RGO stimulated early progression of hair follicles into the anagen phase of the hair cycle and the development of hair follicles, thereby enhancing hair regeneration in a mouse model [67]. In the same study, pure forms of compounds found in RGO, including linoleic acid and β-sitosterol, also produced similar effects on hair regrowth, suggesting that these components of RGO contribute to its ability to promote hair growth. Increased expression of Wingless-type integration-site (Wnt)/β-catenin, sonic hedgehog (Shh)/glioma-associated oncogene homolog (Gli) pathway-related proteins such as β-catenin, phospho-glycogen synthase kinase 3 beta, lymphoid enhancer-binding factor 1, smoothened, Gli1, cyclin D1, and cyclin E indicate that activation of the Wnt/β-catenin and Shh/Gli pathways are required for the hair-growth-promoting activities of RGO, linoleic acid, and β-sitosterol. In addition, RGO and its pure constituent compounds increased the levels in the skin of the growth factors vascular endothelial growth factor (VEGF) and insulin-like growth factor (IGF-1) which are believed to be involved in hair development [67].

In a mouse model of testosterone-induced androgenic alopecia, we showed that a topical application of RGO, linoleic acid, and β-sitosterol induced hair regrowth in a manner comparable to finasteride, an oral drug for treatment of alopecia [83]. RGO, linoleic acid, and β-sitosterol induced hair follicles to enter into the anagen phase that had been delayed by testosterone in C57BL/6 mice. In the same study, testosterone-induced down-regulation of the Wnt/β-catenin and Shh/Gli pathways and upregulation of TGF-β were reversed by the presence of RGO and its pure constituents. However, bicycle (10.1.0)tridec-1-ene, another component of RGO, was not effective at restoring hair growth in a model of testosterone-induced androgenic alopecia [83]. In addition, both linoleic acid and β-sitosterol have been found to inhibit 5α-reductase activity [96,97]. These observations suggest that linoleic acid and β-sitosterol contribute to the ability of RGO to promote hair regeneration. However, RGO contains diverse bioactive compounds that may act synergistically to promote hair growth.

In a study not yet published, our laboratory evaluated the safety of RGO and its ability to promote hair growth in human subjects with androgenic alopecia. Within this study, hair density and hair thickness in both male and female volunteers were increased by topical application of RGO formula after 8–12 weeks, with no adverse effects reported (*unpublished data*). These data suggest that RGO is a potent therapeutic agent for dermatological formulations that prevent and treat androgenic hair loss.

### 4.7 Skin health improvement

RGO can potentially boost the quality and appearance of skin. Topical application of RGO contributes to the protection skin from environmental hazards such as UV radiation [67,98]. RGO exhibited an inhibitory effect on activity of tyrosinase, which is responsible for melanin production [98], but was unlikely to inhibit elastase activity [58]. RGO also suppressed melanin production in UVB-treated mouse skin and α-melanocyte stimulating hormone-stimulated B16/F10 cells, possibly through blocking the microphthalmia-associated transcription factor pathway. In addition, 1% RGO prevented collagen degradation and skin aging [98]. Moreover, a clinical study showed that topical application of moisturizer containing 1% RGO improved moisture content of skin in female volunteers aged 20–30 [57]. Oils that easily absorb into the skin are often used in skincare products to enhance skin moisture content and prevent skin dehydration. The anti-aging and sun-protection properties of RGO can reduce a dull skin appearance and promote skin health. Therefore, RGO can be utilized as an ingredient in the formulation of cosmetics for skincare routines.

### 4.8 Other biological activities of RGO

One previous study has demonstrated an immunomodulatory effect of RGO using *Brucella abortus*-infected macrophages and ICR mice [75]. Treatment with RGO effectively interfered with the adhesion/invasion, uptake, and survival of *Brucella abortus* within macrophages. In the same study, oral administration of RGO in mice significantly decreased splenic proliferation of *Brucella abortus* by regulation of immune system function. Similarly, β-sitosterol in RGO has demonstrated to contribute to inhibitory effects of RGO against *B. abortus* infection [99]. In addition, dietary supplementation with 25 mg of lipophilic fraction from red ginseng inhibited collagen- or thrombin-induced platelet aggregation and blood coagulation in rats [27]. In the same study, dietary intake of the lipophilic fraction from red ginseng reduced serum levels of triglyceride, total cholesterol, high-density lipoprotein cholesterol, and low-density lipoprotein cholesterol, suggesting a potential anti-atherogenic role for RGO.

## 5 Safety of RGO

Several studies have been carried out to evaluate the toxicity and safety of RGO. Our study previously showed that a single oral administration of supercritical CO_2_-extracted RGO at 5000 mg/kg in male and female Sprague–Dawley rats did not cause significant changes in body weight, behavioral patterns, clinical signs, and serum levels of ALT and AST, and no deaths were observed throughout the 14 days of post-administration observation [100]. A further study indicated that oral administration of RGO at up to 2000 mg/kg/day for 28 consecutive days did not cause any detectable toxic effect in both sexes of Sprague–Dawley rats, nor did RGO exert mutagenicity [37]. Similarly, oral administration of a hexane extract of red ginseng at a dose of 1000 mg/kg/day for 15 days did not trigger any toxicity or adverse effects on the health of Balb/c-nu female mice [20]. In a clinical study conducted in our laboratory, volunteers received a topical skin application of supercritical CO_2_-extracted RGO (10%) formula every day for 12 weeks, with no adverse effects reported during the clinical trial (*unpublished data*). These findings demonstrate the safety and nontoxicity of RGO, expanding the areas of its potential application.

## 6 Conclusions

RGO extracted from Korean Red Ginseng is attracting a great amount of attention due to its potential benefits for human health. In this review, we have discussed the extraction methods, chemical composition, biological activities, and safety of RGO. RGO contains a high quantity of fatty acids, especially unsaturated fatty acids, and other lipophilic phytochemicals such as phytosterols, polyphenolic compounds, tocopherols, and polyacetylenes that are beneficial to human health. Solvent extraction and SFE are two main means of RGO production; however, we note that the chemical composition of RGO depends upon the extraction method. Various studies, including *in vitro* and *in vivo* experiments and clinical trials, have showed that RGO possesses various biological activities such as antioxidant, anti-inflammatory, anti-cancer, and anti-apoptotic properties, as well as the ability to promote hair growth; these biological activities are likely associated with its chemical composition. In addition, RGO is generally safe and nontoxic. Based on its value to nutritional and therapeutic applications, RGO is proposed as a novel ingredient for the development of various nutritional, cosmetic, and pharmaceutical products. Our review also suggests that further studies are needed to evaluate novel biological activities and the underlying relationship between the chemical composition and bioactive functionality of RGO.

## Declaration of competing interest

The authors declare no competing interests.

## Nomenclature

ALT: Alanine transaminase
AP-1: Activator protein 1
AST: Aspartate transaminase
CAT: Catalase
COX-2: cyclooxygenase-2
DPPH: 2,2-diphenyl-1-picrylhydrazyl
HO-1: Heme oxygenase-1
Gli: glioma-associated oncogene homolog
GPx: Glutathione peroxidase
IGF-1: insulin-like growth factor 1
iNOS: inducible nitric oxide synthase
IL: interleukin
KEAP1: Kelch-like ECH-associated protein 1
NF-κB: Nuclear factor κB
NQO1: NAD(P)H quinone oxidoreductase 1, Nrf2, Nuclear factor E2-related factor 2
MAPK: Mitogen-activated protein kinase
LPS: Lipopolysaccharide
ORAC: Oxygen radical absorbance capacity
PARP: poly (ADP-ribose) polymerase
RNS: Reactive nitrogen species
ROS: Reactive oxygen species
RGO: Red ginseng oil
SFE: Supercritical fluid extraction
Shh: Sonic hedgehog
SOD: Superoxide dismutase
TGF-β: Transforming growth factor beta
TNF-α: Tumor necrosis factor alpha
UV: Ultraviolet
VEGF: Vascular endothelial growth factor
Wnt: Wingless-type integration-site
